# Secondary psoas abscess after an open cholecystectomy and a common bile duct exploration

**DOI:** 10.1016/j.ijscr.2019.08.026

**Published:** 2019-08-31

**Authors:** Marlon Uriel Alvarado, Cristhian Alejandro Colindres, Luis José Pinto, Cintia G. Padilla, Carlos E. Reyes

**Affiliations:** a Emergency Department, Hospital San Francisco, Juticalpa, Honduras; b Pediatrics Department, Hospital Santa Teresa, Universidad Nacional Autonoma de Honduras, Comayagua, Comayagua, Colonia Lopez, Honduras; c Surgery Deparment, Hospital Escuela Universitario, Universidad Nacional Autonoma de Honduras, Tegucigalpa, Francisco Morazán, Colonia Kennedy primera entrada, Honduras

**Keywords:** Psoas abscess, Cholecystectomy, Complication, Case report

## Abstract

**Background:**

A psoas abscess presents a diagnostic challenge, due to its rarity, non-specific clinical manifestations and wide range of etiologies.

**Case report:**

We describe a case of a secondary psoas abscess containing *Staphylococus aureus* following open cholecystectomy and common bile duct exploration in a woman who had no history or evidence of comorbidity.

**Conclusions:**

Two possible causes of this abscess are performance of the Kocher maneuver during the surgical procedure and external contamination. This case adds cholecystectomy and cholecystitis to the list of known causes of psoas abscesses.

## Highlights

• Psoas abscesses are rare, with a worldwide incidence of only 12 new cases per year.
• Kocher maneuver increases risk of perforation and consequent inoculation of the retroperitoneal space with microorganisms.
• External contamination may occur either through use of contaminated instruments or inadequate asepsis of the surgical area.

## 1 Case report

A 44-year-old woman presented to the emergency department with a 12-day history of fever and abdominal pain radiating to the epigastrium. She had a previous diagnosis of cholelithiasis, but no other pathological and personal antecedents. Her physical examination revealed jaundice, a temperature of 38 °C and pain in the right hypochondrium. A positive Murphy’s sign was elicited. Laboratory studies showed a white blood cell count of 6300 / mm3; hematocrit 39.1%; hemoglobin 12.8 g / dL; and platelets 314,000 cells / mm3; Total bilirubin: 5.1 mg / dL; ALP: 284 IU / L; SGOT: 88 IU / L; and SGPT: 169 IU / L. Ultrasound revealed a distended gallbladder with thick walls containing stones. The extrahepatic bile duct was dilated and contained stones. She was diagnosed with acute cholecystitis and choledocholithiasis, and admitted to the surgery department for a surgical intervention and postoperative care.

An open cholecystectomy and a bile duct exploration via choledochotomy were performed. The surgical findings were: distended gallbladder with thick walls and a dilated bile duct. Both the gallbladder and the bile duct contained stones. A choledocorraphy was performed and a closed suction drain (Hemovac®) was placed in the liver bed. The patient was managed with analgesics and antibiotics (ceftriaxone 2 g IV) for 24 h until discharge.

**Fig. 1:**
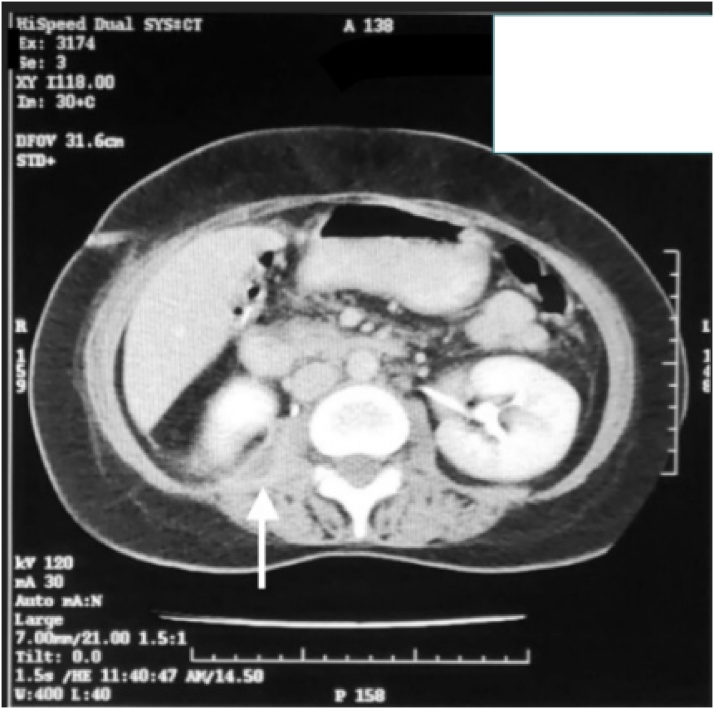
CT showing abdominal collection in the right psoas muscle.

Six days after hospital discharge, the patient returned to the Emergency Department with a 3-day history of a subjective fever and dysuria. Palpation of the suprapubic region revealed slight pain. The hemogram showed leukocytosis of 11,400 cells/mm3, with predominant neutrophils. Urinalysis revealed a high white blood cell count and a positive leukocyte esterase. The patient was evaluated by the surgical team who found a healing surgical wound with a functional drain and distinct biliary material, ruling out the existence of associated surgical complications. After taking a urine sample for culture, empiric ambulatory antibiotic therapy was established Fig. 1.

Three days later the patient returned with a fever of 39.5 C° accompanied by chills, pain on right flank palpation and positive bilateral renal percussion. A new blood count revealed an elevation of the WBC count to 13,200 / mm3 with a left shift. The urinalysis showed no abnormalities. The urine culture, taken 3 days earlier, showed growth of *Escherichia coli* resistant to cephalosporins and fluoroquinolones, and sensitive to aminoglycosides, carbapenemics and trimethoprim-sulfamethoxazole. Hospital admission was indicated. Antibiotic coverage was changed to amikacin (1 g IV).

The patient showed marked improvement in her general condition and a complete resolution of her urinary symptoms in the following 48 h. However, she continued with a fever of 39.5 C°. She was re-evaluated by the Internal Medicine Service, reporting the persistence of pain in the right flank, so a new abdominal ultrasonography was performed, which ruled out intra-abdominal fluid collection and confirmed the correct placement of the drainage system. A broad-spectrum antibiotic coverage with Imipenem (500 mg IV) was started.

To further investigate the cause of the patient’s continuing fever, abdominal computed tomography was undertaken. It revealed diffuse inflammation of the right iliopsoas muscle with a 15 ml fluid collection. A diagnosis of psoas abscess was made. Given the persistence of fever for 72 h despite conservative management, percutaneous drainage of the abscess was performed. Purulent material was obtained. Culture of the material revealed *Staphylococcus aureus* sensitive to carbapenems, vancomycin, ceftriaxone, ciprofloxacin and moxifloxacin. Following the percutaneous drainage, the patient showed marked improvement, and complete cessation of fever. After 7 days of hospitalization she was discharged home with moxifloxacin (400 mg).

## 2 Discussion

Psoas abscesses can be classified as primary and secondary. Primary abscesses originate from hematogenous or lymphatic dissemination from a distant site and are typically caused by monobacterial infections [1,2]. *S. aureus* was found to be the most commonly isolated microorganism in one case series, and was identified in 42.9% of primary abscesses [3]. Secondary abscesses on the contrary, such as the abscess described in this case report, originate by contiguous dissemination of microorganisms from adjacent structures, and can be caused by one or multiple microorganisms, mainly bacteria from the gastrointestinal tract [2,3]. Psoas abscesses are rare, with a worldwide incidence of only 12 new cases per year; the majority in tropical and developing countries. Cases associated with surgical interventions and open cholecystectomy are exceptionally rare, reported in less than 1% of patients [[4], [5], [6]].

An important question raised by this case is how could cholecystectomy cause a psoas abscess, since the liver bed where the gallblader is located is not in direct contact with the psoas muscle. Shonak et al. described a case of retroperitoneal abscess caused by *Haemophilus parainfluenzae* associated with endoscopic retrograde cholangiography (ERCP) and subsequent open cholecystectomy with exploration of the bile duct. That report hypothesized that the infection could have been secondary to small perforations of the retroperitoneum during the sphincterotomy carried out in the ERCP [6]. In our case, the patient underwent an open cholecystectomy with exploration of the bile duct. During this procedure, the Kocher maneuver was performed, an action that pulls on the peritoneum to explore the distal portion of the bile duct in search of stones. We hypothesize that the Kocher maneuver may increase risk of perforation and of consequent inoculation of the retroperitoneal space with microorganisms.

An alternative possibility for causation of the psoas abscess in this case, which is suggested by the isolation of *S. aureus,* an organism more commonly seen in primary psoas abscesses, is that external contamination may have occurred either through use of contaminated instruments or inadequate asepsis of the surgical area.

Despite its rarity, this case adds cholecystectomy and / or cholecystitis to the list of known causes of psoas abscesses.

## Sources of funding

No sources of funding to disclose.

## Ethical approval

Our study is exempted from of ethical approval according to our institution regulations.

## Consent

Written informed consent was obtained from the patient for publication of this case report and accompanying images. A copy of the written consent is available for review by the Editor-in-Chief of this journal on request.

## Author contribution

1. Dr. Marlon Uriel Alvarado contributed to: Study concept, data collection, analysis and interpretation. Also, contributed in writing the paper.

2. Dr. Cristhian Alejandro Colindres Data collection, analysis and interpretation. Also in writing the paper

3. Dr. Luis Jose Pinto Garcia contributed to: Study concept, analysis and interpretation and writing the paper.

4. Dr. Cinthia G. Padilla contributed to: Analysis, interpretation and writing the paper.

5. Dr. Carlos E. Reyes Rendon Analysis, interpretation and writing the paper.

## Registration of research studies

None

## Guarantor

Dr. Marlon Uriel Alvarado Rubi, senior researcher of this manuscript.

## Provenance and peer review

Not commissioned, externally peer-reviewed

## Declaration of Competing Interest

No conflicts of interest to disclose
